# Scientific Items

**Published:** 1887-04

**Authors:** 


### SCIENTIFIC ITEMS.

**Insects.**—The views of Dr. Grassi, that insects, especially flies, may be considered as veritable authors of epidemics, and agents in infectious maladies, are strengthened by the investigations of Dr. R. L. Maddox, as explained in a paper read by him to the Royal Microscopical Society.

He believes that the comma bacillus from cultures can pass, in a living state, through the digestive tube of some insects, and, from this cause, insects feeding upon bacilli are likely to become the media of distributing diseases. Instances have been heretofore published of the discovery of living infinitesimal worms carried from one locality to another in the proboscis of the fly, caught after feeding upon substances containing such worms, and examined with the microscope. Such records open up an immense field of possibilities to the imagination of speculative individuals.

**Circulation Not Essential to Life.**—Circulation of vital fluid is not absolutely essential to life. A tooth, an egg, a seed can live without circulation. If an artificial tooth is placed in the jaw, the gum and alveolar process shrink away from it; it will not adhere to the new object, from want of sympathy. But the natural tooth adheres to the surrounding parts, though it is not inclined to ulcerate, suppurate or experience any other mode of being rejected as a foreign body. An egg has life, and maintains it without circulation And a seed placed outside the influence of heat, moisture and atmospheric action, will germinate after a hundred years. A tooth appears to have no membranes and vessels penetrating it, like other forms of bone, nor is it subject, as above stated, to the changes incidental to other living parts; it has no such pores or membranes; yet it will often adhere to and form connection with its cavity, if timely replaced after removal. —*Medical World.*

**Snow.**—In calm weather, when the snow is falling gently, if we catch the flakes upon a piece of black cloth, and examine them with a magnifying-glass, we often find them to possess forms of wonderful beauty. They are generally star-shaped; and, on counting, we find that there are always six rays or points, or else some simple multiple of that number. If we measure the angles of the branching forms, we find that they always measure exactly sixty degrees; in other words, water crystallizes in the hexagonal system, one of the six great systems of crystillization, which includes the well-known mineral calcite or limestone. Like limestone, also, water is said to be dimorphous, sometimes crystallizing in the trimetric system; but this observation has not been fully confirmed. Snow is simply finely crystallized ice. When more coarsely crystallized, or in half-frozen drops of water, it is known as sleet. When precipitated in large masses of ice, we have hailstones, the formation of which has never been satisfactorily explained.

Probably nearly all of our readers are familiar with the appearance of the country when covered with a mantle of freshly fallen snow. but the few who reside in tropical countries may be assured that they have yet to witness one of the most beautiful manifestations of nature.—*Popular Science News.*

**Carboniferous Age.**—The vegetation of the carboniferous age was much more like the present vegetation of the warm Southern climates than any of the growths of to-day. In closing, Professor Dawson spoke of the immense debt we owe the carboniferous age for our coal, much over half of which is generously given to the United States and England. The production of coal in England has now reached 160,000,000 tons per annum, while the United States produces 100,000,000 tons, and the British colonies 5,000,000 per annum. Professor Huxley has curiously described our obligation in one of his papers on this subject. Nature, he said, is never in a hurry, keeping a thing until a use has been found for it. It was given to the sea, but no use was made of it. Exposed on the land, still no use was found for it until the other day, comparatively, when man, the last creature turned out, so to speak, made a fire, and found the black stone would burn. Coal is now essential to every thing. Without it metallurgical operations could not be carried on, and the great wealth of manufactories and railroads would not be in existence. Nature invested in the coal years ago, and now it is paid back, principal and interest. Professor Dawson's version of this would substitute for "nature," the "Author of nature."—*Ibid.*

**A New Anti-Rabies Inoculation.**—"Dr. Fernandez, of Barcelona, claims to have discovered a new vaccine to preserve men and animals from the contagion of rabies. He has collected a great number of observations, showing that dogs bitten accidently by vipers are never affected by rabies, either spontaneously or when bitten by affected animals. He has made certain direct experiments, inoculating dogs with a small quantity of the viper's poison. After inoculation, the animals were ill for four or five days with symptoms of slight fever, prostration, and more or less profound somnolence. He maintains that the animals thus operated on are protected from rabies, and that neither when inoculated with the saliva, nor when bitten by affected animals, do they contract the disease."

**Hermit Crab.**—The hermit crab possesses the wonderful instinct of finding the cast-off shell of some other animal which he occupies. As their size increases, they move out of the old house, and start off in search of a better-fitting one. They sometimes find a desirable residence already occupied by another hermit crab, and then an amusing but desperate fight takes place; and, if victorious, the house-hunter turns out the rightful occupant, and snugly ensconces himself in the shell, while the evicted tenant must move on until he finds another one.—*Pop. S. News.*

